# Metformin: update on mechanisms of action on liver diseases

**DOI:** 10.3389/fnut.2023.1327814

**Published:** 2023-12-14

**Authors:** Gaoyi Ruan, Fangquan Wu, Dibang Shi, Hongxia Sun, Fangyan Wang, Changlong Xu

**Affiliations:** ^1^Department of Gastroenterology, The Second Affiliated Hospital and Yuying Children’s Hospital of Wenzhou Medical University, Wenzhou, China; ^2^Department of Pathophysiology, School of Basic Medicine Science, Wenzhou Medical University, Wenzhou, China

**Keywords:** non-alcoholic liver disease, metformin, cirrhosis, AMPK pathway, drug-induced liver injury

## Abstract

Substantial attention has been paid to the various effects of metformin on liver diseases; the liver is the targeted organ where metformin exerts its antihyperglycemic properties. In non-alcoholic fatty liver disease (NAFLD), studies have shown that metformin affects the ATP/AMP ratio to activate AMPK, subsequently governing lipid metabolism. The latest research showed that low-dose metformin targets the lysosomal AMPK pathway to decrease hepatic triglyceride levels through the PEN2-ATP6AP1 axis in an AMP-independent manner. Metformin regulates caspase-3, eukaryotic initiation factor-2a (eIF2a), and insulin receptor substrate-1 (IRS-1) in palmitate-exposed HepG2 cells, alleviating endoplasmic reticulum (ER) stress. Recent observations highlighted the critical association with intestinal flora, as confirmed by the finding that metformin decreased the relative abundance of *Bacteroides fragilis* while increasing *Akkermansia muciniphila* and *Bifidobacterium bifidum*. The suppression of intestinal farnesoid X receptor (FXR) and the elevation of short-chain fatty acids resulted in the upregulation of tight junction protein and the alleviation of hepatic inflammation induced by lipopolysaccharide (LPS). Additionally, metformin delayed the progression of cirrhosis by regulating the activation and proliferation of hepatic stellate cells (HSCs) via the TGF-β1/Smad3 and succinate-GPR91 pathways. In hepatocellular carcinoma (HCC), metformin impeded the cell cycle and enhanced the curative effect of antitumor medications. Moreover, metformin protects against chemical-induced and drug-induced liver injury (DILI) against hepatotoxic drugs. These findings suggest that metformin may have pharmacological efficacy against liver diseases.

## 1 Introduction

Metformin is a classical oral hypoglycemic agent derived from a leguminous caudex named *Galega officinalis* (1). Its antihyperglycemic properties were first discovered in 1918, and in 1957, Jean Sterne successfully used it to treat diabetes mellitus (2). Given its substantial safety and efficacy, metformin is indicated as a first-line medication for type 2 diabetes treatment (3).

**Figure 1:**
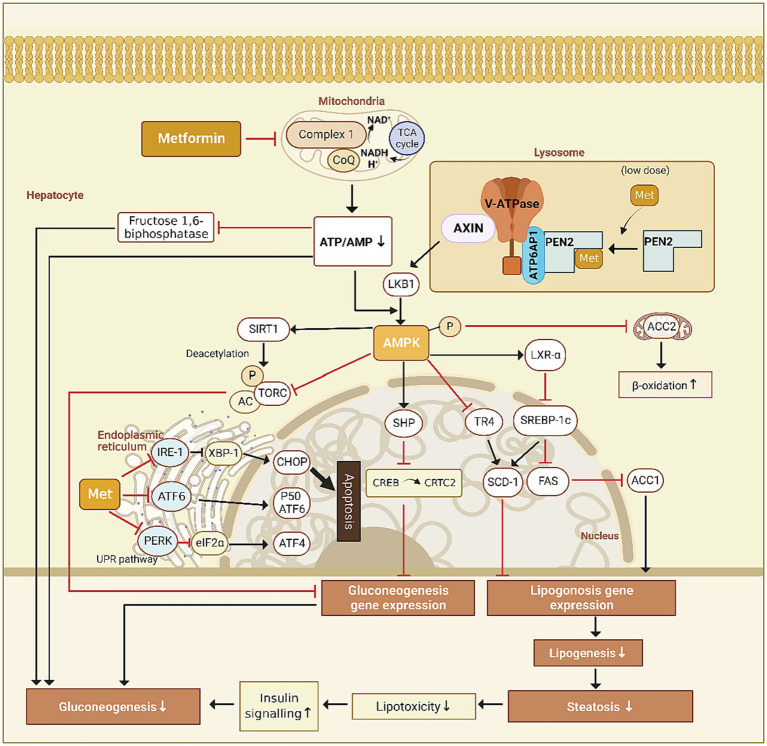
Proposed mechanisms of action for metformin.

The liver is the primary target organ of metformin, which inhibits hepatic glucose output through the AMPK signaling pathway in the following ways. (1) Metformin upregulates the small hetero-dimer partner through the AMPK signaling pathway, which interacts directly with the cAMP-response-element-binding factor (CREB) to block the recruitment of CREB to CREB-regulated transcription co-activator 2 (CRTC2), downregulating the expression of gluconeogenic genes (4); (2) Through the AMPK signaling pathway, metformin upregulates the expression of the hepatic deacetylase sirtuin 1 (SIRT1), which deacetylates CRTC2 and promotes its ubiquitinated degradation, downregulating the expression of gluconeogenic genes (5). The lysosomal v-ATPase-regulator complex is a common activator for AMPK and mTORC1, acting as a switch between catabolism and anabolism (6). However, it is yet unknown how metformin activates AMPK (Figure 1).

Later, it was found that metformin activates AMPK through the lysosomal pathway and disrupts metabolic processes such as ATP synthesis through oxidative phosphorylation. Mechanistically, it acts on vacuolar H + -ATPase (v-ATPase) and promotes the translocation of AXIN/LKB1 onto the surface of lysosome to form complex with v-ATPase-regulator and then dissociates raptor and mTOR, leading to AMPK activation and turning off the activity of mTORC1, a master regulator for anabolic pathways (7).

Ma et al. synthesized a photoactive metformin probe to identify potential direct targets. By promoting intestinal GLP-1 secretion through the lysosomal AMPK pathway, low-dose metformin lowers blood glucose via PEN2 in a AMP-independent manner. However, even though metformin inhibits hepatic gluconeogenesis, researchers found that low-dose metformin did not behave in such a way, based on pyruvate tolerance tests and the quantification of gluconeogenic genes. Furthermore, metformin exerted two supplementary beneficial effects through the AMPK-determined PEN2-ATP6AP1 axis, specifically diminishing hepatic lipid accumulation in mice liver and prolonging the lifespan of *Caenorhabditis elegans*, in addition to this postprandial glucose reduction (8).

In recent years, several lines of evidence revealed various biological effects of metformin in addition to improving glucometabolic status, including anti-inflammation, antioxidation, antitumor, and anti-fibrosis; metformin holds considerable potential to prevent or treat non-alcoholic fatty liver disease (9), cirrhosis (10), hepatic malignancies (11), drug-induced or chemical-induced liver injury (12), and other liver diseases. This article reviews its role and underlying mechanisms in liver disease treatment.

## 2 NAFLD

NAFLD is a clinical-pathological syndrome not directly linked to alcohol or other etiologies. It is characterized by excessive lipid accumulation in hepatocytes, one of the most common types of chronic liver disease worldwide (13, 14). NAFLD includes the benign NAFLD and the more severe non-alcoholic steatohepatitis (NASH), which carries a higher risk of progression to severe liver disease (15, 16). Lipotoxicity theory explains the progression of NAFLD (17). According to the theory, large amounts of free fatty acids (FFAs) ectopically accumulate in the liver, which causes impaired hepatocytes’ ability to output FFAs into triglycerides (TGs) and subsequent toxic effects. This phenomenon only increases insulin resistance (18) and also promotes disease progression (17). Metformin can relieve NAFLD by reducing lipotoxicity in the following ways.

Recently, a consensus has been reached to replace the term NAFLD with metabolic dysfunction–associated steatotic liver disease (MASLD) following a modified Delphi process (19). MASLD is defined as the presence of hepatic steatosis and at least one of five cardiometabolic risk factors. This change in terminology aims to increase disease awareness, reduce stigma, and accelerate the development of drugs and biomarkers for the benefit of patients. However, the proposed nomenclature concept has limitations in classifying subtypes of NAFLD due to insufficient data. It is important to highlight that metabolic syndrome and its components are strongly associated with NAFLD/MASLD (20). Therefore, metformin, a drug targeting various factors related to metabolic syndrome, such as fasting plasma glucose levels and waist circumference, may also alleviate NAFLD in the following ways.

### 2.1 Metformin regulates lipids metabolism by activation of AMPK

Several studies dealt with the downstream signaling pathway of AMPK. Nevertheless, none identified a specific target for AMPK activation by metformin before Ma et al. found that lysosomal PEN2 is recruited to ATP6AP1 subunit upon binding to metformin to regulate v-ATPase, which provides the impetus for the translocation of AXIN and liver kinase B1 (LKB1) to the lysosomal surface to activate AMPK, enabling low concentrations of metformin to utilize the AMP-independent AMPK activation pathway (8).

Metformin activates LKB1 to induce AMPK phosphorylation and inhibits acetyl-CoA carboxylase (ACC), which has several complex effects. Fullerton et al. demonstrated that metformin treatment alters hepatic lipid homeostasis and increases insulin sensitivity by suppressing ACC (21). 103 In fasting, Metformin increases AMPK activity, which increases the activity of LXR-α. LXR-α then suppresses sterol regulatory element binding protein-1c (SREBP-1c, a master regulator of liver lipogenesis gene programme), which in turn suppresses enzymes of lipogenesis [ACC1, fatty acid synthase (FAS) and stearoyl-CoA desaturase 1 (SCD1)], thereby inhibiting lipogenesis (22). AMPK can also directly inhibit ACC2, decrease malonyl Co-A, and activate carnitine palmitoyl transferase 1 (CPT1), and thereby enhancing β-oxidation. In addition, ACC1 (cytosolic) is highly expressed in lipogenic tissues, such as liver and adipose tissue, while ACC2 (mitochondrial membrane) is majorly expressed in oxidative tissues, such as cardiac and skeletal muscle, which are consistent with the functions of ACC1 in lipogenesis and ACC2 in regulating fatty acid β-oxidation (23).

Activated by metformin, AMPK phosphorylates nuclear receptor TR4 at Ser351 to inhibit transcriptional activation of TR4, repressing the expression of TR4-mediated gene for stearyl coenzyme A dehydrogenase-1 (24). It also phosphorylates SREBP-1c at Ser372, which inhibits proteolytic maturation, nuclear translocation of SREBP-1c and the expression of downstream fatty acid synthase gene, thus increasing fatty acid β-oxidation and reducing *de novo* synthesis of TG (25) to prevent hepatocyte steatosis by reducing the accumulation of TG in HepG2 cells (26).

Metformin-induced NAFLD remission could be attributed to the cooperative roles of Kuffer cells (KCs) and hepatocytes, which are mediated by the presence of tristetraprolin (TTP), an mRNA-binding protein. Metformin activates TTP in hepatocytes and KCs through the AMPK/Sirt1 pathway. TTP inhibited TNF-α production in KCs, resulting in a subsequent reduction in hepatocyte necroptosis. Metformin-induced TTP activation inhibited mTORC1 via destabilization of Rheb, which promotes transcription factor-EB nuclear translocation to promote hepatocyte lipophagy, treating obesity-related NAFLD (27).

### 2.2 Metformin alleviates endoplasmic reticulum (ER) stress

ER stress refers to a homeostatic imbalance within the ER caused by external stimulus or changes in the intracellular environment, which further leads to the accumulation of unfolded or misfolded proteins in the ER. It can cause cytotoxic effects and unfolded protein response (UPR) (28). Although proper UPR acts as a protective agent against misfolding, enhancing the ER folding capacity, and degrades misfolded proteins, excessive or persistent UPR can trigger cellular insults, such as the execution of apoptosis, which accelerates NAFLD progression (29). Cell culture and animal studies found that metformin may act to reduce ER stress. Indeed, metformin attenuated palmitate-induced UPR events in RINm5F rat insulinoma cells (30) and HepG2 cells (31), and alleviated hepatic ER stress of Male albino Wistar rats induced by a high-calorie diet (29). The UPR is initiated through three sensors: the protein kinase RNA-like ER kinase (PERK), the inositol requiring enzyme-1 (IRE-1), and the activating transcription factor-6 (ATF-6). Accumulated unfolded proteins bind to immunoglobulin-binding proteins, activating PERK, IRE-1, as well as transcription factor-6 and phosphorylating eIF2a. In response to activation of IRE-1, active ATF6 translocates to the Golgi apparatus, enters the nucleus, and triggers apoptosis through C/EBP homologous protein (CHOP) (29). As reported by Kim et al., metformin significantly suppresses caspase-3 and eIF2a phosphorylation, blocking the induction of the ER stress markers in palmitate-exposed HepG2 cells. The expression of spliced XBP-1 mRNA and cleaved ATF6 was at low levels, suggesting that metformin regulates ATF6 processing, inhibits selected IRE-1 activities, and regulates the expression of pro-apoptotic CHOP (31). Metformin also reversed the palmitate-induced serine phosphorylation of IRS-1, thus attenuating ER stress to inhibit hepatocellular apoptosis resulting from lipotoxicity (30).However, it should be noted that a study by Geng et al. showed that while metformin inhibited palmitate-induced eIF2α phosphorylation in HepG2 cells, it did not downregulate the expression of CHOP, Gadd34, or GRP78, which are the downstream target genes of p-eIF2α (32). This finding suggests that the dephosphorylation of *p*-eIF2α does not lead to deactivation of the downstream UPR signaling pathway, meaning that metformin is not directly related to lower ER stress. The precise process is debated.

### 2.3 Metformin restores mitochondrial function

Lipotoxicity can potentially harm the mitochondrial respiratory chain by accelerating the tricarboxylic acid cycle flux in NAFLD (33, 34). Geng et al. discovered that metformin inhibits the mitochondrial respiratory chain complex to prevent the electron transport from NADH to coenzyme Q, which somewhat lowers mitochondrial basal and maximal respiration and mildly limits ATP synthesis. In response to metformin treatment, primary rat hepatocytes exposed to palmitate experienced a recovery of mitochondrial respiration. Further study showed that palmitate decreased mitochondrial membrane potential and increased cell reactive oxygen species (ROS) by detecting mitochondrial membrane potential. At the same time, metformin restored the membrane potential, reducing mitochondrial proton leak and ROS generation by inducing the expression of superoxide dismutase 2. Thus, the protective effects of metformin on palmitate-induced cell death could result from partially inhibiting mitochondrial complex I, which would restore mitochondrial function and demonstrate the necessity of mitochondrial complex I in retarding or stopping the progression of NAFLD (32). Before this study, the concentrations of metformin used in reports investigating the mechanism of action of metformin varied widely, often at much higher than the blood concentration of the therapeutic dose of metformin. Consequently, research on the mechanism of action of metformin has produced contradictory findings. Emphasis should be given to the importance of proper inhibition of mitochondrial complex I since excessive or insufficient metformin cannot have its intended therapeutic effect. Studies will be needed to determine the appropriate metformin dose for use in NAFLD therapy.

### 2.4 Metformin downregulates non-coding RNA associated with NAFLD

In recent years, the critical role of non-coding RNA such as microRNA, lncRNA, and circRNA has become recognized in NAFLD (35–37). Dysregulation of non-coding RNA disrupts the gene regulatory network, leading to metabolic syndrome and related diseases. MicroRNAs are small non-coding RNAs involved in post-transcriptional gene expression regulation by binding to the 3′-untranslated region of target mRNAs and inhibiting their expression (38). A broad spectrum of NAFLD has aberrantly enhanced miR-34a expression in humans and mice (39). By coordinating the regulation of lipid metabolism, mRNAs regulate NAFLD development and progression. Silencing miR-34a led to an initially increased expression of hepatic peroxisome proliferation-activated receptor-α (PPARα), SIRT1, then PPARα and SIRT1 activated the AMPK pathway. PPARα and pAMPKα 1 increased fat oxidation and improve the steatosis finally (40). *In vitro* experiments conducted by Xu et al.showed that the ablation of hepatocyte miR-34a resulted in the inhibition of intestinal lipid absorption and hepatic TG synthesis, as well as a decrease in inflammation, ROS production, apoptosis, and an induction of hepatic fatty acid oxidation. Furthermore, the *in vivo* experiments showed that miR-34a inhibitors reduced hepatic levels of TG and FFAs. These findings were further supported by a reduction in hepatic expression of genes associated with bile acid synthesis (CYP7A1 and CYP8B1) and fatty acid synthesis (SREBP-1c, ACC1, and FAS), while genes involved in fatty acid oxidation (PPARα, CPT1, CPT2, and PDK4) were upregulated, which may account for the mechanism by which inhibition of miR-34a expression protects against the development of steatohepatitis (41). Others reported that miR-34a was upregulated in MCD-fed mice, and following treatment with metformin, this effect attenuated miRNA expression (42). However, no further studies were conducted. Thus, the study mentioned above may provide insight into the potential mechanism by which metformin halts the advancement of NAFLD.

### 2.5 Metformin reshapes the gut microbiota and intestinal barrier function

The occurrence and development of NAFLD are associated with impaired intestinal flora metabolism and intestinal permeability. Several lines of evidence suggest that metformin improves intestinal microflora composition and intestinal barrier dysfunction (43–45), suggesting that this pathway might be essential for metformin against NALFD. The FXR, a bile acid-activated nuclear receptor prominently expressed in the liver and intestine, regulates the expression of genes involved in cholesterol and bile acid homeostasis, hepatic lipogenesis, and inflammation, in addition to maintaining the intestinal barrier integrity, preventing bacterial translocation and preserving the gut microbiota eubiosis. Based on previous studies, the imbalance of FXR is one of the critical mechanisms in the development of NAFLD (46). Metformin treatment decreased the abundance of species of *Bacteroides fragilis* and its bile salt hydrolase activity in the intestines of individuals with T2D, increasing the levels of the bile acid glycoursodeoxycholic acid to inhibit intestinal FXR signaling (47). Another study showed that metformin is associated with higher relative abundance of mucin-degrading *Akkermansia muciniphila* and *Bifidobacterium bifidum* (43, 48, 49) Both strains protected against NAFLD by activating hepatic FXR, suppressing intestinal FXR expression, modulating the gut microbiota, and improving intestinal mucosal permeability (50). The administration of *A. muciniphila* restored metabolic disturbances in diet-induced obese mice. These imbalances include heightened adiposity and metabolic endotoxemia. Damage to the intestinal barrier is an early core event and an essential mechanism for NAFLD development. Li et al. (51) discovered that *A. muciniphila* given by daily oral gavage in high-fat diet-fed mice partially restored the thickness of the mucin layer and upregulated epithelial tight junction proteins, specifically zona occludens 1 and occludin. These alterations ultimately lead to a reduction in the influx of pro-inflammatory LPS into the systemic circulation, resulting in a subsequent decrease in the expression of hepatic inflammatory markers. Furthermore, *A. muciniphila* raised the level of gut endocannabinoids, pivotal in controlling inflammation, maintaining intestinal barrier integrity, and encouraging the release of gut peptides from intestinal L-cells. Experimental evidence from *in vivo* and *in vitro* studies substantiated the endocannabinoid system’s role in regulating the gut-intestinal barrier via a mechanism dependent on the CB1 receptor (52). A cross-sectional metagenomic study conducted across three countries revealed a positive correlation between the utilization of metformin and the presence of short-chain fatty acid-producing bacteria, including *Bifidobacterium bifidum*, *Butyrivibrio*, *Megasphaera*, and an operational taxonomic unit of *Prevotella* (48, 53). Acetate promoted by carbohydrate transporter in bifidobacterial mediates anti-apoptotic and anti-inflammatory responses in the host colonic epithelium, protecting the host against lethal infection (54). Cani et al. (55) demonstrated that manipulating gut microbiota in mice favoring the *Bifidobacterium* spp. significantly improves gut permeability and obesity-induced hepatic inflammatory phenotype in a glucagon-like peptide-2-dependent way. These findings suggest that microbes at least partially mediate the positive effects of metformin on host metabolism. The findings further illustrate the diversified modes of metformin in NAFLD treatment with direct effects on the liver and indirect actions via extrahepatic pathways.

## 3 Hepatic fibrosis and cirrhosis

Hepatic cirrhosis is a diffuse liver injury characterized by degeneration and necrosis of hepatocytes, replacement of normal liver tissue by fibrotic tissues and nodules, and progressive loss of liver function (56). Hepatic fibrosis is an essential pathological process in which liver lesions develop into cirrhosis (57), characterized by excessive accumulation of connective tissue and abnormal formation of fibrous septum, the primary component of which is type I collagen produced by activated HSCs (58). HSC activation is the classical mechanism for promoting liver fibrosis (59). In response to injury and inflammatory factors, HSCs transform into highly proliferative and migratory myofibroblasts, upregulate the expression of α-SMA, a sign of HSC activation, and produce a large amount of collagen and other extracellular matrix (ECM), leading to fibrosis (60, 61). Metformin improves outcomes by treating NAFLD/NASH to delay the progression to cirrhosis and inhibits hepatic fibrosis (62). Mounting evidence has indicated that it can control the activation and proliferation of HSC through various signal pathways.

### 3.1 Metformin activates AMPK to inhibit the platelet-derived growth factor (PDGF) pathway

The PDGF signaling pathway is one of the most characteristic pathways in HSC activation. PDGF-BB, a subtype of the PDGF family, is induced by the most potent stimulator of HSC growth and intracellular signal transduction. PDGF induces the activation of extracellular signal-regulated kinase and Akt/mTOR pathways, which are serine/threonine protein kinases that are critical in cell growth, differentiation, proliferation, migration, and survival. Metformin activated AMPK to regulate PDGF-BB-induced phenotypic changes in HSC activation (63, 64). Adachi et al. demonstrated that metformin inhibited PDGF-induced phosphorylation of AKT, FoxO1 and mTOR/p70S6K (two downstream targets of the phosphatidylinositol 3-kinase/AKT pathway), resulting in the inhibition of HSC proliferation and migration, the reduction of extracellular matrix secretion consisting of *α*-SMA, type I collagen and fibronectin, which leads to inhibition of fibrosis (64, 65).

### 3.2 Metformin inhibits the TGF-β1/Smad3 pathway

Another mechanism of HSC activation is the cooperation of TGF-β1-related signaling pathways and other signals, including ROS, PDGF, and connective tissue growth factors (66). Smad3 is the primary downstream target. In human and rat HSC lines, activated AMPK Inhibited TGF-β-Induced fibrotic responses of HSCs by regulating transcription co-activator P300 without altering TGF-β-induced Smad3 phosphorylation, nuclear localization, and Smad3 promoter binding activity. This regulation manifested as increasing proteasomal degradation to induce p300 downregulation but not attenuating p300 expression (67). Fan. et al. studied CCl4-exposed mice and found that metformin reduced the expression of TGF-β1 and inhibited the TGF-β1/Smad3 pathway, thereby inhibiting the expression of fibroblast genes and fibroblast markers such as α-SMA and E-cadherin directly mediated by Smad3 (68–70). Xiao et al. (71) established a model of myocardial fibrosis. They found that metformin can directly bind to TGF-β1 through an AMPK-independent pathway, reducing its binding probability to TGF-β1 type II receptor rather than binding force and leading to the reduction of downstream signal transduction, which might explain how metformin inhibits TGF-β1 signaling.

### 3.3 Metformin inhibits the succinate-GPR91 pathway

Succinate is an intermediate product of the tricarboxylic acid cycle, which can be converted into fumaric acid by succinate dehydrogenase. In addition to its critical role in energy metabolism, it acts as an extracellular signaling molecule that binds to and activates its specific G-protein-coupled receptor (GPR91), a receptor in HSCs whose role in hepatic fibrosis remains unclear. In an *in vitro* and *in vivo* study, Li. et al. found that HSCs cultured and treated directly with succinate or succinate dehydrogenase inhibitors increased the expression of GPR91 and the expression of α-SMA, TGF-β, and type I collagen, which are markers of the fibrotic response. These findings suggested that succinate accumulation and GPR91 overexpression are pathological features of hepatic fibrosis (72). Nguyen et al. (10) found that in mice fed with a methionine-choline-deficient diet, the expression of α-SMA and GPR91 is enhanced, while metformin administration reduced overexpression in the liver, ameliorating steatohepatitis and liver fibrosis in mice. In addition, metformin significantly reduced the enhanced HSC activation, proliferation, and migration processes induced by succinate and inhibited the expression of inflammatory cytokines (e.g., IL-6 and TGF-β1). These findings suggest that metformin acts as an antifibrotic agent by inhibiting the succinate-GPR91 pathway.

## 4 HCC

Hepatoma is a common malignant digestive system tumor with significant morbidity and mortality. HCC, which makes up 70–90% of the pathological types of primary liver cancer, is now the second most common cause of cancer death worldwide. Epidemiological evidence suggests that metformin can reduce the risk of cancer in diabetic patients (73), while HCC is one of the cancer types with the most significant reduction in incidence after the application of metformin (74). Here, we discuss how metformin exerts its underlying anti-cancer effects through the following pathways.

### 4.1 Metformin inhibits the growth of liver cancer cells

Activation of the mTOR signaling pathway, a branch downstream of AMPK, plays an essential role in the occurrence and progression of cancer, primarily hepatocellular carcinoma. The AMPK activation by metformin enhances the binding of DEPTOR and mTOR by increasing DEPTOR production and inhibiting proteasomal degradation, thereby interfering with the downstream target p70-S6 kinase and ribosomal protein S6 and inhibiting the proliferation of hepatocellular carcinoma cells (75, 76). Metformin-activated AMPK also reduces the degradation of intracellular caspase-3 protein by inhibiting the proteasome, inducing apoptosis in tumor cells (77, 78).

### 4.2 Metformin interferes with energy metabolism of liver cancer cells

The impact of metformin on tumor energy metabolism may be what causes it to have antitumor properties. Increased glycolysis is a characteristic of many tumors (79, 80). Glycolysis provides tumor cells with the energy they need for basal activities, and its metabolites can be employed to synthesize biomacromolecules and NADPH necessary for developing and invading cancerous cells. A study found that a hypoglycemia-metformin combination restrains tumor growth by activating glycogen synthase kinase 3β (GSK3β) downstream of PP2A. Mechanically, metformin inhibits CIP2A, a PP2A suppressor. At the same time, hypoglycemia upregulates B56δ, the PP2A regulatory subunit, resulting in the formation of a PP2A-B56δ active complex with a high affinity toward GSK3β to specifically activate the PP2A-GSK3β axis, leading to a decline in the pro-survival protein MCL-1, thereby interfering with glycolysis in hepatocellular carcinoma cells (81). Due to the rapid growth of tumor tissue, local hypoxia is ubiquitous in tissues, including liver cancer cells. Composed of *α* and *β* subunits, the heterodimeric hypoxia-inducible factor 1 (HIF-1), a critical transcription factor that regulates the response of tumor cells to hypoxia, can cause the transcription of a variety of genes to make it possible for the tumor cells to tolerate oxygen environment (82). HIF-1α is upregulated in many malignant tumors and reflects the tendency of tumor metastasis and poor prognosis (83). Metformin inhibits mitochondrial respiration by inhibiting respiratory chain complex I and reduces oxygen consumption to improve hypoxia (84), thereby promoting the ubiquitination of HIF-1α and the degradation of HIF-1α by the proteasome. The stability of HIF-1α and the translocation of the α subunit to the nucleus to bind to HIF-1β is reduced, resulting in the decrease of its ability to activate the cancer-promoting target genes glucose transporter 1 and carbonic anhydrase IX, exerting the role of inhibiting liver cancer progression (85–87). The effect of metformin on promoting degradation of HIF-1α can also inhibit the allosteric activator of phosphofructokinase-1 6-phosphofructose-2-kinase/fructose-2, 6-bisphosphatase 3 (PFKFB3), downregulating the expression of PFK1 mediated by PFKFB3 to inhibit glycolysis and cancer cell proliferation (88).

### 4.3 Epigenetic modification

MiRNAs play critical roles in liver cancer (89). Among these RNAs, miR-21 is oncogenic for HCC. Liu et al. (90) found that miR-21 is highly upregulated in liver cancer cells; the reduced expression of miR-21 inhibited the proliferation and migration of liver cancer cells through the phosphate and tension homology and triggered G2/M cell cycle arrest in cancer cells. Miyoshi et al. used metformin to treat liver cancer cells *in vivo* and *in vitro* and found that metformin inhibits human HCC cell proliferation and arrests the G1 cell cycle by suppressing the cell cycle-related molecules via alteration of miRNAs. Fifty-one differentially expressed miRNAs were identified, among which the let-7 family (let-7a, let-7b, and let-7e) was upregulated in metformin-treated liver cancer cells (91). The human let-7 family contains 13 members and is a class of tumor suppressor miRNAs (92). The upregulated miRNAs in these studies included miRNAs overexpressed in HCC (e.g., miR-21). The investigators speculated that this phenomenon might be related to altered survival responses in malignant cells. Additional investigations are required to determine whether each miRNA reflects the cause or effect of metformin treatment.

### 4.4 Metformin with cytotoxic therapy

Several studies explored the possibility of combining metformin with cytotoxic therapy for liver cancer; several lines of evidence suggest that metformin enhances the anti-cancer effect of these agents. WP631 is a structural analog of doxorubicin that exerts anti-cancer effects by inducing cell apoptosis. Its molecular mechanism includes activating NF-κB and increasing Bax, p53, and caspases-3, 8, and 9. Sliwinska et al. found that metformin can accelerate the death of cancer cells induced by WP631 and reduce the dose of WP631, which may be related to a significant increase in the level of NF-κB in liver cancer cells (93). These authors also discovered that metformin enhanced the anti-hepatoma effect of tubulin depolymerization inhibitor epothilone A by enhancing its pro-apoptotic effect and increasing levels of NF-κB (94).

## 5 Drug-induced and chemical-induced liver injury (DILI)

DILI refers to many forms of liver injury during medication exposure, resulting from metabolite hepatotoxicity or poor drug tolerance in specific patient populations. Given that the incidence is increasing yearly in China and Western countries because of substance abuse (95). DILI is the most common cause of acute liver failure (96). Chemical hepatotoxicants cause chemical-induced liver injury and are similar to DILI in mechanism. Hence, these two diseases will be discussed together.

### 5.1 Metformin ameliorates APAP-induced liver injury

In the Western world, APAP overdose is the most common cause of DILI and acute liver failure (97). Excessive APAP is converted to N-acetylbenzoquinone imine (NAPQI) by hepatic cytochrome P450 2E1. It depletes reduced glutathione in mitochondria, and the remaining NAPQI then reacts to form covalent links with cellular biological macromolecules, especially proteins, resulting in mitochondrial damage and necrotic cell death. This process increases ROS production, triggering JNK phosphorylation and sustained JNK activation, which contributes to liver cell death (98). Kim et al. (12) found that metformin protects against APAP overdose-evoked hepatotoxicity via growth arrest and DNA damage 45β (GADD45β)-dependent JNK regulation. Metformin can increase the expression of growth arrest and GADD45β to inhibit the phosphorylation of mitogen-activated protein kinase kinase 4, inhibiting JNK phosphorylation to protect hepatocytes from oxidative damage. However, another study indicated that metformin does not inhibit JNK activation or mitochondrial JNK translocation; metformin significantly reduced APAP protein adducts in mitochondria. Additionally, metformin inhibits mitochondrial respiratory chain complex I, which lowers proton leak and ROS generation in hepatocyte mitochondria, thereby reducing hepatocyte apoptosis to treat DILI (99).

### 5.2 Metformin ameliorates DILI induced by other chemicals

Metformin protects against chemical-induced liver injury. D-galactosamine increases the level of LPS and TNF-α in hepatocytes, causing acute liver injury. Metformin can reduce inflammatory indicators such as myeloperoxidase and malondialdehyde by activating the classic AMPK signaling pathway to inhibit apoptosis induced by LPS and TNF-α, thereby alleviating liver injury (100, 101). Moreover, arsenic trioxide, a chemotherapy agent used to treat promyelocytic leukemia, causes liver damage by generating ROS, while metformin can inhibit mitochondrial respiratory chain complex I and increase NAD+/NADH ratio, protecting against ATO damage. In addition, metformin can prevent carbon tetrachloride-induced hepatotoxicity, possibly associated with the increase of glutathione in hepatocyte mitochondria (102).

## 6 Conclusion

Given recent advances in understanding metformin’s role in activating AMPK, attenuating lipotoxicity, optimizing gut microbiome, and regulating HSCs and liver cancer cells, the drug has potential therapeutic applications in several liver diseases. However, the actual clinical application of metformin for various *in vivo* disorders needs to be approached with caution due to limited relevant clinical research, and the correlation of dose must be considered when designing new metformin-based therapies. More research in these fields will advance our knowledge of metformin as a novel treatment for several liver diseases.

## Author contributions

GR: Writing – review & editing, Writing – original draft. FW: Writing – original draft. DS: Writing – original draft, Writing – review & editing. HS: Writing – original draft. FW: Writing – original draft. CX: Writing – review & editing.
